# Genome-scale metabolic modeling underscores the potential of *Cutaneotrichosporon oleaginosus* ATCC 20509 as a cell factory for biofuel production

**DOI:** 10.1186/s13068-020-01838-1

**Published:** 2021-01-06

**Authors:** Nhung Pham, Maarten Reijnders, Maria Suarez-Diez, Bart Nijsse, Jan Springer, Gerrit Eggink, Peter J. Schaap

**Affiliations:** 1 Laboratory of Systems and Synthetic Biology, Wageningen University & Research, Wageningen, the Netherlands; 2 Food and Biobased Research and AlgaePARC, Wageningen University and Research, Wageningen, the Netherlands; 3 Bioprocess Engineering and AlgaePARC, Wageningen University and Research, Wageningen, the Netherlands; 4 Department of Ecology and Evolution, University of Lausanne, Swiss Institute of Bioinformatics, 1015 Lausanne, Switzerland

**Keywords:** Genome-scale metabolic model, *Cutaneotrichosporon oleaginosus* ATCC 20509, Lipid accumulation, Crude glycerol, Biodiesel production, Flux balance analysis, Oleaginous yeast

## Abstract

**Background:**

*Cutaneotrichosporon oleaginosus* ATCC 20509 is a fast-growing oleaginous basidiomycete yeast that is able to grow in a wide range of low-cost carbon sources including crude glycerol, a byproduct of biodiesel production. When glycerol is used as a carbon source, this yeast can accumulate more than 50% lipids (w/w) with high concentrations of mono-unsaturated fatty acids.

**Results:**

To increase our understanding of this yeast and to provide a knowledge base for further industrial use, a FAIR re-annotated genome was used to build a genome-scale, constraint-based metabolic model containing 1553 reactions involving 1373 metabolites in 11 compartments. A new description of the biomass synthesis reaction was introduced to account for massive lipid accumulation in conditions with high carbon-to-nitrogen (C/N) ratio in the media. This condition-specific biomass objective function is shown to better predict conditions with high lipid accumulation using glucose, fructose, sucrose, xylose, and glycerol as sole carbon source.

**Conclusion:**

Contributing to the economic viability of biodiesel as renewable fuel, *C. oleaginosus* ATCC 20509 can effectively convert crude glycerol waste streams in lipids as a potential bioenergy source. Performance simulations are essential to identify optimal production conditions and to develop and fine tune a cost-effective production process. Our model suggests ATP-citrate lyase as a possible target to further improve lipid production.

## Background

Microbial lipids produced by oleaginous yeasts are promising sources for oleochemical replacements of hazardous petrochemicals in fuels and chemicals [1, 2]. For the establishment of an economical bio-based utilization, cost-effective production is key. Of the fewer than 30 known oleaginous yeasts, the top five most studied species are *Yarrowia lipolytica*, *Rhodotorula glutinis*, *Rhodosporidium toruloides*, *Cutaneotrichosporon oleaginous*, and *Lipomyces starkeyi* [1]. The profile of lipids and fatty acids produced by these yeasts varies, but under natural conditions they can, on average, accumulate lipids up to 40% of their weight [3, 4]. A lipid content of up to 70% can be obtained if in the presence of a carbon source, an essential nutrient is depleted [4]. Under such conditions, excess carbon will be re-routed to storage compounds, being lipids in oleaginous yeasts [3, 5]. Nitrogen limitation, often referred to as a high C/N ratio has been shown to be the most efficient inducer of such lipid accumulation [4].

As input materials are one of the main contributors to production cost [6], for an economically feasible process, a natural capacity for high lipid biosynthesis may not be enough. Oleaginous yeasts are able to use a range of alternative sugars for lipid production (Table 1). Among them, *C. oleaginosus* appears to be one of the most accommodating and is able to grow in a wide range of industrially interesting operational conditions such as in food waste and municipal wastewater streams [7], whey permeate [8], office paper production waste streams [9, 10], spent yeast lysate from brewery industry and crude glycerol from biodiesel production [11, 12]. Lipid production by this yeast has been studied for at least two decades [4, 8, 13–16] and when growing on crude glycerol, *C. oleaginosus* can accumulate more lipid content than many other yeasts, microalgae or molds (Table 1). Owing to these advantages, *C. oleaginosus* is flagged as one of the most cost-effective and versatile cell factories for de novo lipid production [1, 17]. Especially when the inexpensive waste product from biodiesel production, crude glycerol, is becoming abundantly available, this organism could play a major role in further upcycling of the biodiesel process, as lipids derived from *C. oleaginosus* grown on glycerol have high concentrations of monounsaturated fatty acids (MUFA) [18]. MUFAs are excellent biodiesel components due to their low temperature fluidity and oxidative stability [18].

**Table 1:** Lipid yields obtained by oleaginous yeasts

Organism	Yield^a^	Carbon source	Reference
*Yarrowia lipolytica*	0.27	Glucose	[80]
*Yarrowia lipolytica*	0.10	Crude glycerol	[81]
*Rhodosporidium toruloides*	0.29	Lignocellulosic hydrolysates	[82]
*Rhodotorula glutinis*	0.18	Molasses	[83]
*Lipomyces starkeyi*	0.24	Glucose	[84]
*Cutaneotrichosporon oleaginosus*	0.22	Glucose	[85]
*Cutaneotrichosporon oleaginosus*	0.29	Whey permeate	[8]
*Cutaneotrichosporon oleaginosus*	0.27	Crude glycerol	[18]

^a^g-lipid/g-substrate

*C. oleaginosus* is a basidiomycete yeast of the *Tremellomycetes* class and recently added to the *Cutaneotrichosporon* genus [19]. Taxonomically, it has been reclassified and renamed several times as *Apiotrichum curvatum*, *Cryptococcus curvatus*, *Trichosporon cutaneum*, *Trichosporon oleaginosus*, and *Cutaneotrichosporon curvatum* [11, 12]. In this study, we will refer to it as *Cutaneotrichosporon oleaginosus* ATCC20509 [20, 21]. The yeast can metabolize a wide range of oligo- and monomeric sugars such as cellobiose, xylose, sucrose, lactose, and glucose [22]. Xylose is efficiently metabolized via the phosphoketolase pathway and partly via the pentose phosphate pathway [11, 23]. Both pathways produce pyruvate as intermediate for further metabolic processes [11].

Despite many efforts spent on studying this yeast, its use for the production of lipids from glycerol is still far from optimized [1, 10, 24]. Recently, a response surface method was used to design experiments to optimally explore the relationship between the carbon-to-nitrogen ratio in the medium and lipid production and to guide the design of optimal production media for *C. oleaginosus* ATCC20509 [25]. However, the translation from the genotype to a (preferred) phenotype [i.e. high lipid production], is typically a multi-factorial process depending on the growth medium, culture conditions, strain specificity and the interplay among these factors. Hence, a predictive constraint-based, genome-scale model of metabolism (GEM), along with genetic accessibility tools [26] will provide new avenues towards reaching the full potential of *C. oleaginosus* ATCC 20509 as a lipid producer [11].

By drawing upon a thorough functional re-annotation of its genome, we have built a GEM for *C. oleaginosus* ATCC 20509. The model is named *i*NP636_*Coleaginosus*_ATCC20509, expanding the usual naming convention for GEMs [27] by including information on the organism considered to enhance recognition. Subsequently, the model was used to investigate optimal lipid production in glycerol.

## Results and discussion

### Annotation

One of the major bottlenecks in eukaryotic genome annotation is the identification of exon–intron boundaries. In this regard, transcriptome data can provide a good basis for predicting introns. We therefore collected transcriptome data (RNAseq) of *C. oleaginosus* ATCC 20509 from two conditions and used it to structurally annotate genome sequence MATS00000000.1 of *C. oleaginosus* ATCC 20509 [28].

BRAKER1 [29] predicted 7861 protein coding genes. Of these, 7474 genes are directly supported by RNAseq with more than 50 read counts per million (CPM). Among the protein-coding genes, 5621 proteins with functional protein domains (Pfam release 31) and 2358 with a full unique Enzyme Commission (EC) number could be predicted. A summary is provided in Table 2. A complete annotation is provided in Additional file 1.

**Table 2:** Genome annotation results for *Cutaneotrichosporon oleaginosus* ATCC 20509

Annotation features	Results
Genome size (Mbp)	19.86
No. of protein coding genes	7861
Protein length (median no. of amino acids)	409
Gene length (median bp)	1708
Transcript length (median bp)	2460
No. of genes with intron	6891
Proteins with at least one functional domain assigned	5621
No. of predicted (partial) EC’s	627
No. of predicted (full) unique EC’s	1072
Proteins with a predicted (full) EC’s	1778

### Lipid synthesis pathways

**Fig. 1:**
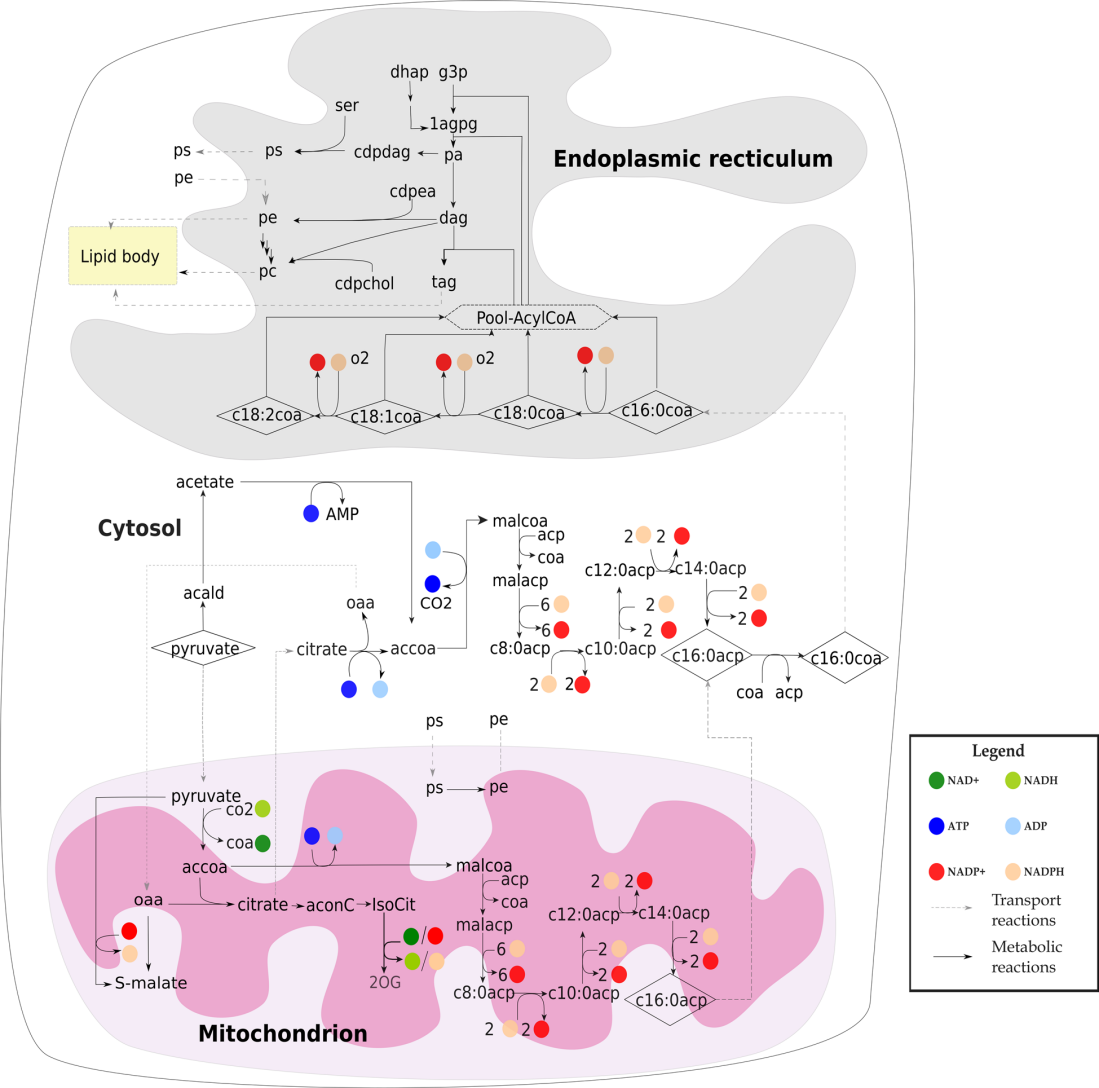
Lipid synthesis pathway in *C. oleaginosus* ATCC 20509. 1agpg-1-Acyl-sn-glycero-3-phosphoglycerol; 2OG-2-oxoglutarate; acald-acetaldehyde; accoa-acetyl-CoA; aconC-cis-aconitate; acp-acyl carrier protein; c8:0acp-octanoyl acyl carrier protein; c10:0acp-decanoyl acyl carrier protein; c12:0acp-dodecanoyl acyl carrier protein; c14:0acp-tetradecanoyl acyl carrier protein; c16:0acp-Hexadecanoyl acyl carrier protein; c16:0coa-Hexadecanoyl-coa; c18:0coa-Octadecanoyl-coa; c18:1coa-oleoyl-CoA; c18:2coa-linoleoyl coA; cdpdag-CDP-Diacylglycerol; cdpea-CDP-ethanolamine; cdpchol-CDP-choline; coa-coenzyme A; dag-diacylglycerol; g3p-glyceraldehyde 3-phosphate; oaa-oxaloacetate; IsoCit-Isocitrate; malacp-malonyl acyl carrier protein; malcoa-malonyl-CoA; pa-phosphatidate; pc-phosphatidylcholine; pe-phosphatidylethanolamine; ps-phosphatidylserine; ser-serine; tag-triacylglycerol

*C. oleaginosus* ATCC 20509 metabolizes sugars by using standard central metabolic pathways including glycolysis, pentose phosphate pathway and the citric acid (TCA) cycle. The yeast metabolizes xylose via the phosphoketolase pathway and partly via the pentose phosphate pathway [11, 23]. These pathways provide the precursors and energy required for lipid biosynthesis. Lipid biosynthesis can be divided into three steps: formation of fatty acids, synthesis of triacylglyceride (TAG), and synthesis of phospholipids (Fig. 1).

### Formation of fatty acids

In yeasts, fatty acids can derive from either a de novo synthesis pathway or from hydrolysis of complex lipids and delipidation of proteins, and from hydrolysis of external fatty acids sources [30]. De novo fatty acid synthesis generally occurs in the cytosol [4], and in some cases, in the mitochondrion [31]. This pathway produces saturated fatty acids up to 16 C atoms while further elongation and desaturation takes place in the endoplasmic reticulum (ER) [4, 32]. The process is catalyzed by the multi-enzyme fatty acid synthetase complex (FAS) [4]. We found multiple genes, g2870.t1, g5734.t1, g570.t1 and g5733.t1, (Additional file 1) that together encode this enzyme complex in *C. oleaginosus* ATCC 20509. The overall process of fatty acid synthesis in *C. oleaginosus* ATCC 20509 (Fig. 1) can be simplified as follows:

$$ {\text{ATP}} - {\text{citrate lyase }}\left( {{\text{ACL}}} \right):{\text{Citrate}}\, + \,{\text{ATP}}\, \to \,{\text{oxaloacetate}}\, + \,{\text{acetyl}} - {\text{CoA}}\, + \,{\text{ADP}}\, + \,{\text{P}}_{i} . $$

$$ {\text{Acetyl}} - {\text{CoA Carboxylase }}\left( {{\text{ACC}}} \right):{\text{acetyl}} - {\text{coA}}\, + \,{\text{CO}}_{{2}} \, + \,{\text{ATP}}\, \to \,{\text{malonylCoA}}\, + \,{\text{ADP}}\, + \,{\text{P}}_{i} . $$

$$ \begin{gathered} {\text{Fatty acid synthetase }}\left( {{\text{FAS}}} \right):{\text{acetyl}} - {\text{CoA}}\, + \,{\text{7 malonyl}} - {\text{CoA}}\, + \,{\text{14 NADPH }} \hfill \\ \, + \,{\text{14 H}}^{ + } \, \to \,{\text{palmityl}} - {\text{CoA}}\, + \,{\text{14 NADP}}^{ + } \, + \,{\text{7 CoA}}\, + \,{\text{7 CO}}_{{2}} . \hfill \\ \end{gathered} $$

For the formation of unsaturated fatty acids (C16:1, C18:1, and C18:2) a fatty acid desaturase is required [33]. A single gene, g3345.t1, was predicted to encode this enzyme in *C. oleaginosus* ATCC 20509.

### Synthesis of triacylglyceride and phospholipids

Like in other oleaginous yeast, the process of triacylglyceride (TAG) synthesis in *C. oleaginosus* ATCC 20509 starts with the formation of phosphatidic acid (PtdOH) from glycerol-3-phosphate either through the glycerol-3-phosphate or the dihydroxyacetone phosphate pathway [31, 34] (Fig. 1). PtdOH is subsequently converted to diglyceride which later by addition of one acyl-CoA becomes triacylglyceride.

The main phospholipids in *C. oleaginosus* ATCC 20509 are phosphatidylcholine, phosphatidylethanolamine and phosphatidylserine [35]. They are synthesized from the CDP-diacylglycerol (CDP-DAG) and the Kennedy (or CDP-choline) pathways [32, 36] (Fig. 1).

We provide more details of the reconstructed *C. oleaginosus* lipid synthesis pathway in Additional file 2.

### Features of the model

The GEM for *C. oleaginosus* was constructed using the well-curated GEM iNL895 [37] of the oleaginous model organism *Y. lipolytica* as a template. A template-based approach is often more efficient than starting from scratch however, as the use of a template could limit the scope of the specific GEM, an in-depth *C. oleaginosus*-specific curation of the here important target pathways, i.e. the fatty acid and lipid synthesis was performed. Of the 895 genes underlying the *Y. lipolytica* model, de novo genome annotation followed by manual curation led to the identification of 636 orthologs genes in *C. oleaginosus* ATCC 20509 that were used to generate the *i*NP636_*Coleaginosus*_ATCC20509 GEM. A full list of orthologs is provided in Additional file 3. Both models cover the central carbon and lipid metabolism but, accounting for the differences in lipid and fatty acid profiles in these two organisms, in lipid formation there are differences in the number of isoenzymes involved. A comparison of enzymes involved lipid metabolism of *C. oleaginosus* ATCC20509, *Y. lipolytica* and the non-oleaginous model yeast, *Saccharomyces cerevisiae* is shown in Table 3.

**Table 3:** Enzymes involved in lipid metabolism in *Saccharomyces cerevisiae* model, iNL800 [70], *Yarrow lipolytica* model, iNL895 [37] and *Cutaneotrichosporon oleaginosus* ATCC 20509 model, *i*NP636_*Coleaginosus*_ATCC20509 (this study)

EC	Function	*S. cerevisiae* (iNL800)	*Y. lipolytica* (iNL895)	*C. oleaginosus* ATCC 20509
EC 6.2.1.1	Acetyl-coenzyme A synthetase 1	Y	–	–
EC 6.2.1.1	Acetyl-coenzyme A synthetase 2	Y	Y	Y
EC 1.3.1.9	Fatty acid synthase subunit beta	Y	Y (2)	Y
EC 2.3.1.86	Fatty acid synthase subunit alpha	Y	Y (2)	Y
EC 2.7.7.41	Phosphatidate cytidylyltransferase	Y	Y (2)	Y
EC 2.7.8.11	CDP-diacylglycerol–inositol 3-phosphatidyltransferase	Y	Y	Y
EC 2.7.8.8	CDP-diacylglycerol–serine O-phosphatidyltransferase	Y	Y	Y
EC 2.7.1.30	Glycerol kinase	Y	Y	Y (2)
EC 1.1.1.8	Glycerol-3-phosphate dehydrogenase (NAD( +))	Y (2)	Y	Y (2)
EC 2.3.1.51	Probable 1-acyl-sn-glycerol-3-phosphate acyltransferase	Y	Y (2)	Y
EC 2.3.1.20	Diacylglycerol O-acyltransferase	Y	Y	Y
EC 2.3.1.158	Phospholipid:diacylglycerol acyltransferase	Y	Y	Y
EC 3.1.1.3	Triacylglycerol lipase	Y (3)	Y (2)	Y (2)
EC 2.3.1.26	Acyl-CoA:sterol acyltransferase	Y	Y	Y
EC 1.14.19.1	Acyl-CoA desaturase	Y	Y	Y
EC 1.14.19.6	∆12 Fatty acid desaturase	–	Y	Y
EC 1.3.3.6	Acyl-coenzyme A oxidase	Y	Y (3)	Y
EC 2.3.1.16	3-ketoacyl-CoA thiolase, peroxisomal	Y	Y	Y (2)
EC 2.3.3.8	ATP-citrate lyase, subunit a	–	Y	Y
EC 2.3.3.8	ATP-citrate lyase, subunit b	–	Y	Y
EC 1.1.1.38	NAD-dependent malic enzyme, mitochondrial	Y	Y	Y
EC 6.4.1.2	Acetyl-CoA carboxylase	Y	Y	Y

Y indicates the presence of the enzyme-encoding gene, (−) indicates the absence of the enzyme-encoding gene. Number of isoenzymes is indicated in brackets

Compared to *S. cerevisiae* there are few differences. *S. cerevisiae* lacks an ATP:citrate lyase and does not have the gene encoding for a ∆12 fatty acid desaturase, which introduces the second double bond in the biosynthesis of 18:3 fatty acids. In *S. cerevisiae*, acetyl-CoA is produced from acetyl-coenzyme A synthetase encoded by two distinct genes *ACS1* and *ACS2* representing the “aerobic” and “anaerobic” forms of acetyl-coenzyme A synthetase, respectively [38]. In *C. oleaginosus* ATCC 20509 and *Y. lipolytica*, there is only one acetyl-coA synthase gene, similar to *ACS2* in *S. cerevisiae*.

**Fig. 2:**
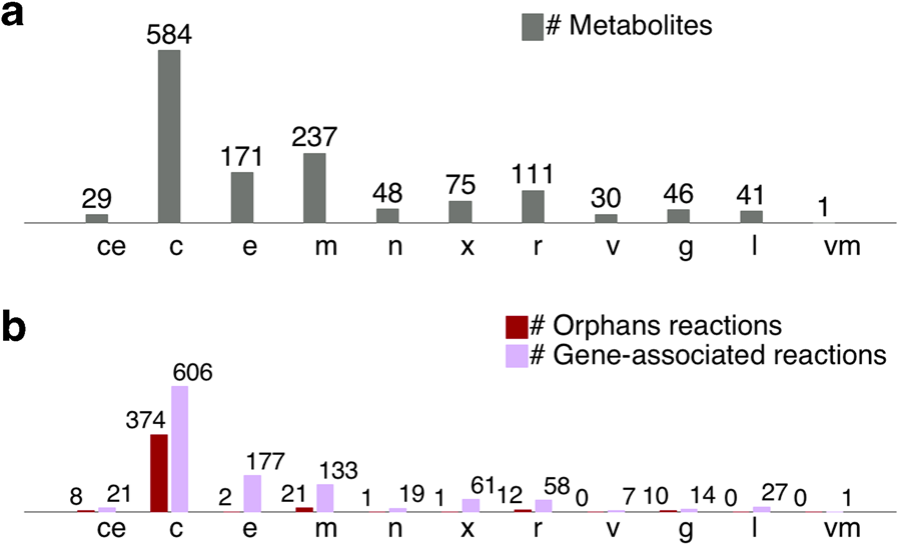
Distribution of (**a**) metabolites and (**b**) reactions among compartments in *i*NP636_Coleaginosus_ATCC20509. Orphan reactions are exchange reactions, transport reactions, spontaneous reactions and reactions with no associated catalyzing genes. *c*-cytosol, ce-cell envelope, *e*-extracellular, *g*-Golgi, l-lipid particle, *m*-mitochondrion, *n*-nucleus, *r*-endoplasmic reticulum, *v*- vacuole, vm- vacuolar membrane, *x*-peroxisome

After curation, the final GEM (*i*NP636_*Coleaginosus*_ATCC20509) contains 1553 reactions, 1373 metabolites, 636 genes, and 11 compartments: cytoplasm, Golgi apparatus, cell envelope, endoplasmic reticulum, mitochondrion, nucleus, peroxisome, vacuolar membrane, vacuole, extracellular and lipid particle representing lipid droplets (Table 4 and Fig. 2).

**Table 4:** Characteristics of *i*NP636_*Coleaginosus*_ATCC20509 model

Categories	Features
Total reactions	1553
Gene-associated reactions	1142 (84%)
Exchange reactions	189
Transport reactions	486
Metabolic reactions	878
Total metabolites	1373
Unique metabolites	786
Genes	636
Compartments	11

Unique metabolites indicate species regardless of compartment

### Biomass synthesis reaction

The biomass synthesis reaction included in the model represents the formation of the main building blocks required for growth of the target organism [39, 40]. Application of growth-limiting nutrients however, may induce large variations in biomass composition. Model flux distributions can be very sensitive to such changes, compromising the predictive accuracy of the metabolic model [40].

The biomass composition of *C. oleaginosus* ATCC 20509 was shown to vary along with the C/N ratio in the medium [8, 25, 41], as in nitrogen-limiting conditions excess carbon is converted to lipids.

**Fig. 3:**
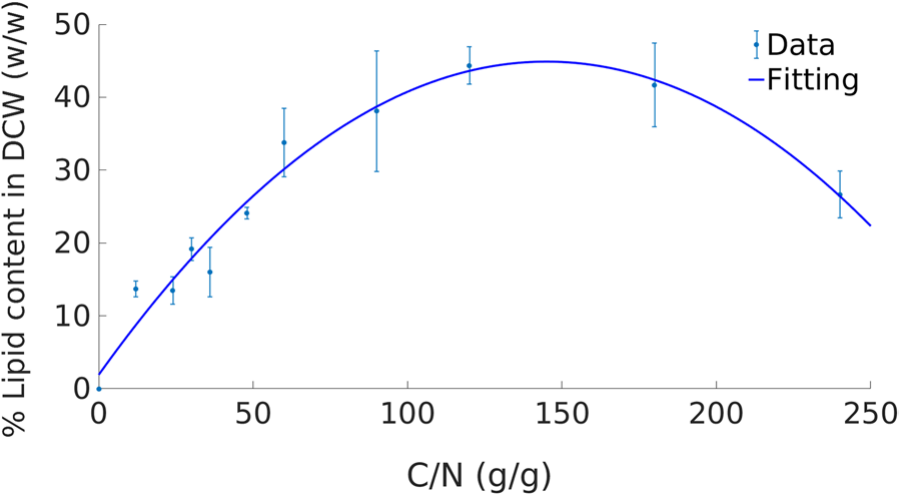
Lipid content in *C. oleaginosus* ATCC 20509 dry cell weight (DCW) at different C/N ratio. In vitro data were obtained from [25]. Fitting line corresponds to *y* = − 0.002*x^2^ + 0.59*x + 1.9

Experimental data show an increase in lipid content with increasing C/N ratio [25] until a maximum is observed at C/N ratio of 120 g/g (Fig. 3). The link between lipid content and C/N ratio can be approximated by a quadratic relationship, as shown in Fig. 3. In addition to lipids, the biomass content of protein and carbohydrate also varies with the C/N ratio [41]. Here, we model weight fraction of biomass that corresponds to carbohydrates (given by *w*_*C*_), proteins (*w*_*P*_) and total lipids (*w*_*TL*_) in the biomass using: *w*_*C*_ *+* *w*_*P*_ + *w*_*TL*_ = 0*.*95 *biomass*. The remaining 5% of the biomass weight is assigned to RNA, DNA, minerals and cofactor content. As these represent minor quantities in the biomass, their coefficients are assumed to be constant.

Upon nitrogen starvation, the yeast cells start accumulating intracellular sugars [41] as short-term energy storage [11, 42]. These intracellular sugars will be then converted to long-term energy storage in form of lipid droplet [41]. Furthermore, nitrogen depletion leads to a decrease in protein content as proteins are used as nitrogen source. No changes in carbohydrate profile in the cell wall under nutrient shortage conditions has been reported [11]. Therefore, we assume that nitrogen depletion will lead to a maximum carbohydrate content in the cell and that the excess carbon will be rerouted for lipid synthesis. Data from [8] at a relatively low C/N ratio (2.8) suggest 11% as a reasonable and conservative estimate for this weight fraction. Combining this expression and the relationship in Fig. 3a biomass synthesis reaction for nitrogen starvation can be dynamically built for any C/N ratio (Additional file 4).

The amount of lipids in the biomass reaction varies along with the C/N ratio, however the lipid composition does not change. TAGs still make up 90% of total lipid in *C. oleaginosus* ATCC 20509 [4] and phospholipids for the remaining 10% [4]. The phospholipids, phosphatidylserine, phosphatidylethanolamine and phosphatidylcholine are added with equal weights. Finally, the fatty acid content of lipids (25% hexadecanoic (C16:0), 10% octadecanoic acid (C18:0), 57% oleic acid (C18:1), and 7% linoleic acid (C18:2) [4, 5]) were also considered to be stable.

### Lipid production and growth in C. oleaginosus ATCC 20509

#### Effect of the C/N ratio on lipid production

**Fig. 4:**
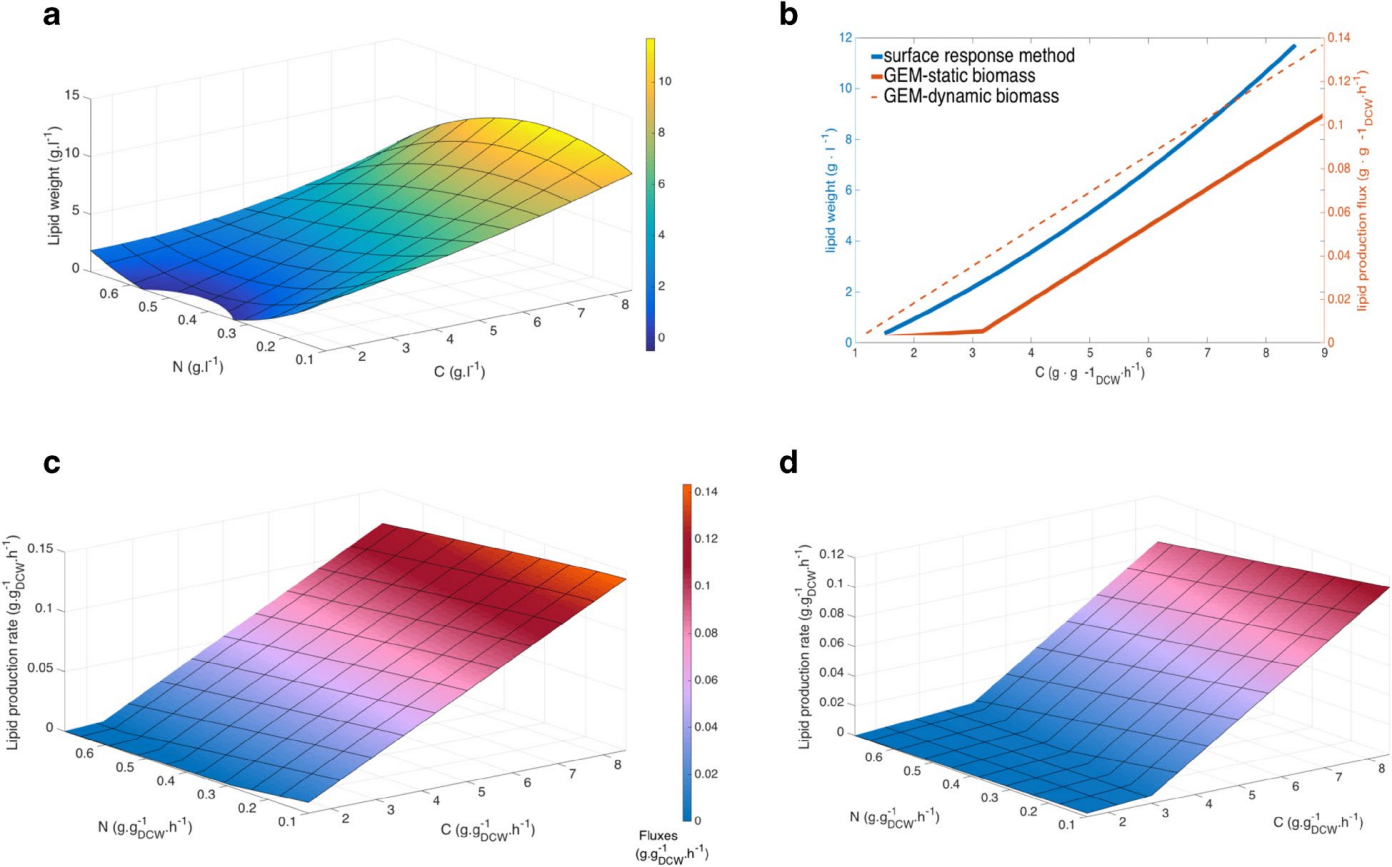
Simulations of impact of C/N ratio on lipid production by *C. oleaginosus* ATCC 20509. **a**: Simulation using the surface response model [25], **b**: A comparison of lipid production at a fixed N concentration at 0.3 (g/l) between surface response method in [25] and *i*NP636_Coleaginosus_ATCC20509 using a standard biomass (static) and condition-specific (dynamic) biomass. **c**: *i*NP636_Coleaginosus_ATCC20509 simulation using the proposed condition-specific biomass objective function, **d**: *i*NP636_Coleaginosus_ATCC20509 simulation using a standard biomass objective function. Glucose is used as a carbon source as was used in the response method in [25]

We compared simulation results from our model, *i*NP636_*Coleaginosus*_ATCC20509, with simulations obtained from the response surface method [25] using either a fixed standard or a condition specific biomass objective function. The results are presented in Fig. 4.

When the condition-specific biomass objective function is applied (Fig. 4b) GEM predictions are better aligned with predictions obtained with the response surface method in [25] (Fig. 4a) underpinning the crucial role of high C/N ratios in lipid production.

### Effect of the carbon source on lipid production and growth

**Fig. 5:**
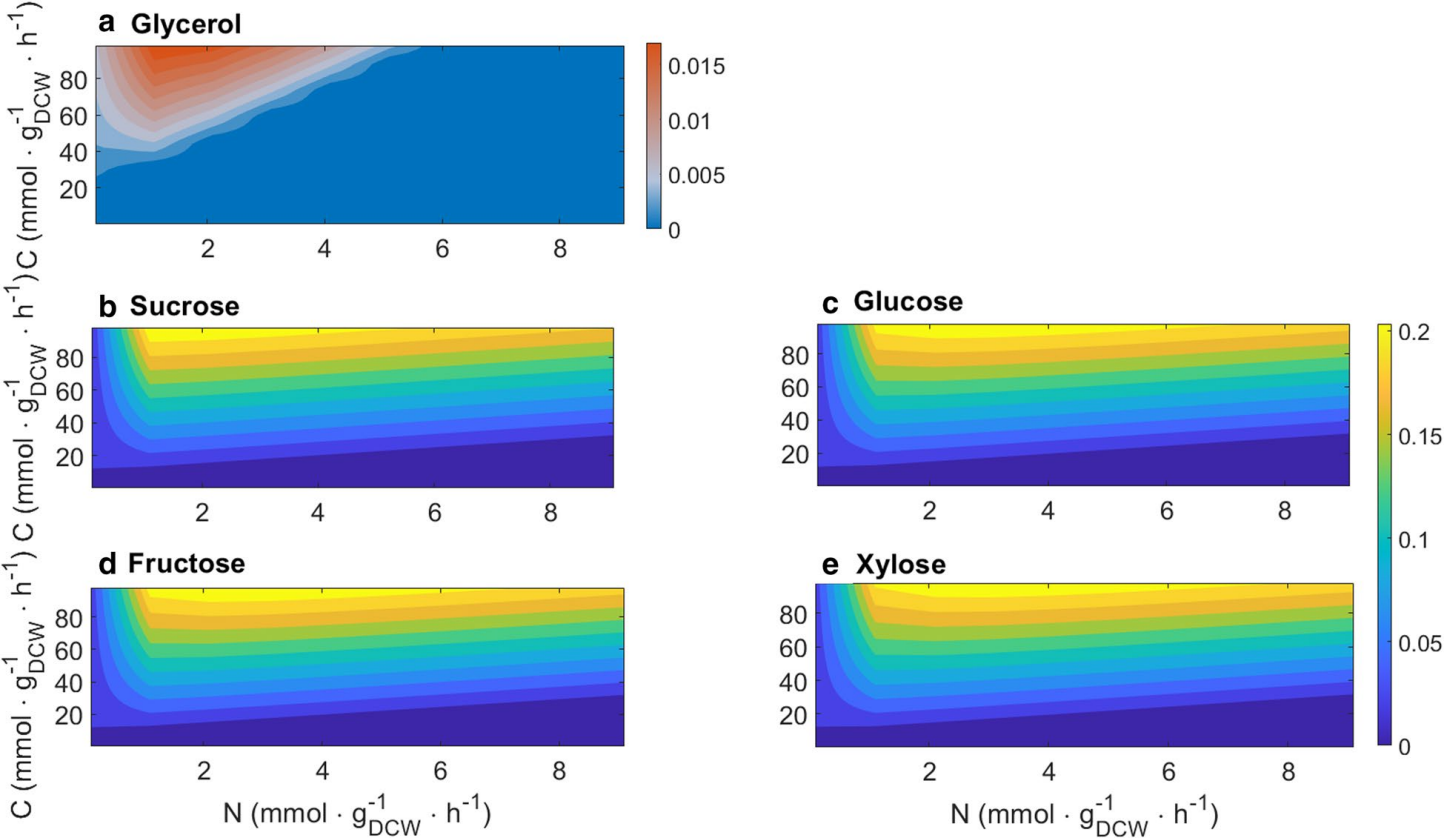
In silico growth rate on different carbon sources. *X* and *Y* axis start from 0.1. Color bars indicate growth rate (h^−1^)

Carbon sources have been shown to have different effects on growth and lipid production in oleaginous yeast [1, 8]. *C. oleaginosus* ATCC 20509 is able to grow on glycerol, sucrose, glucose, fructose, ethanol or xylose as sole carbon source [11, 25] and in silico growth was evaluated on these sources (Fig. 5).

Overall, except for ethanol growth was predicted in all tested carbon sources. In our in silico experiment, uptake rates were adjusted for each carbon source to guarantee the same C-mol was provided. On all tested carbon sources the model predicted favorable growth in nutrient-rich conditions. Comparable growth rates were obtained in sucrose, glucose, fructose and xylose (Fig. 5). A lower growth rate was obtained for glycerol while no growth was obtained when ethanol was used as sole carbon source.

Effects of 5-carbon sugars, i.e. xylose, 6-carbon sugars, i.e. glucose and fructose, and of sucrose on growth in *C. oleaginosus* ATCC 20509 have been studied extensively, and results vary among these studies. According to [25], comparing fructose, glucose, xylose and sucrose, *C. oleaginosus* ATCC 20509 grows the fastest in fructose, the slowest in sucrose while there is no significant difference between glucose or xylose. According to [26], xylose is favored over glucose for biomass generation. These differences can be due to various factors such as pH, temperature, oxygen, dilution rate, and fermentation modes across experiments. When growing in different fermentation modes, i.e. batch, fed batch, and continuous fermentation, the microorganisms are subjected to differences in environment, substrate availability and by-product concentration [43]. In addition, different carbon sources may have different uptake rates. These factors can result in different growth rates, biomass and by-product accumulation. In this study, we simulated the process in continuous fermentation and assumed the same uptake rate for all carbon sources.

**Fig. 6:**
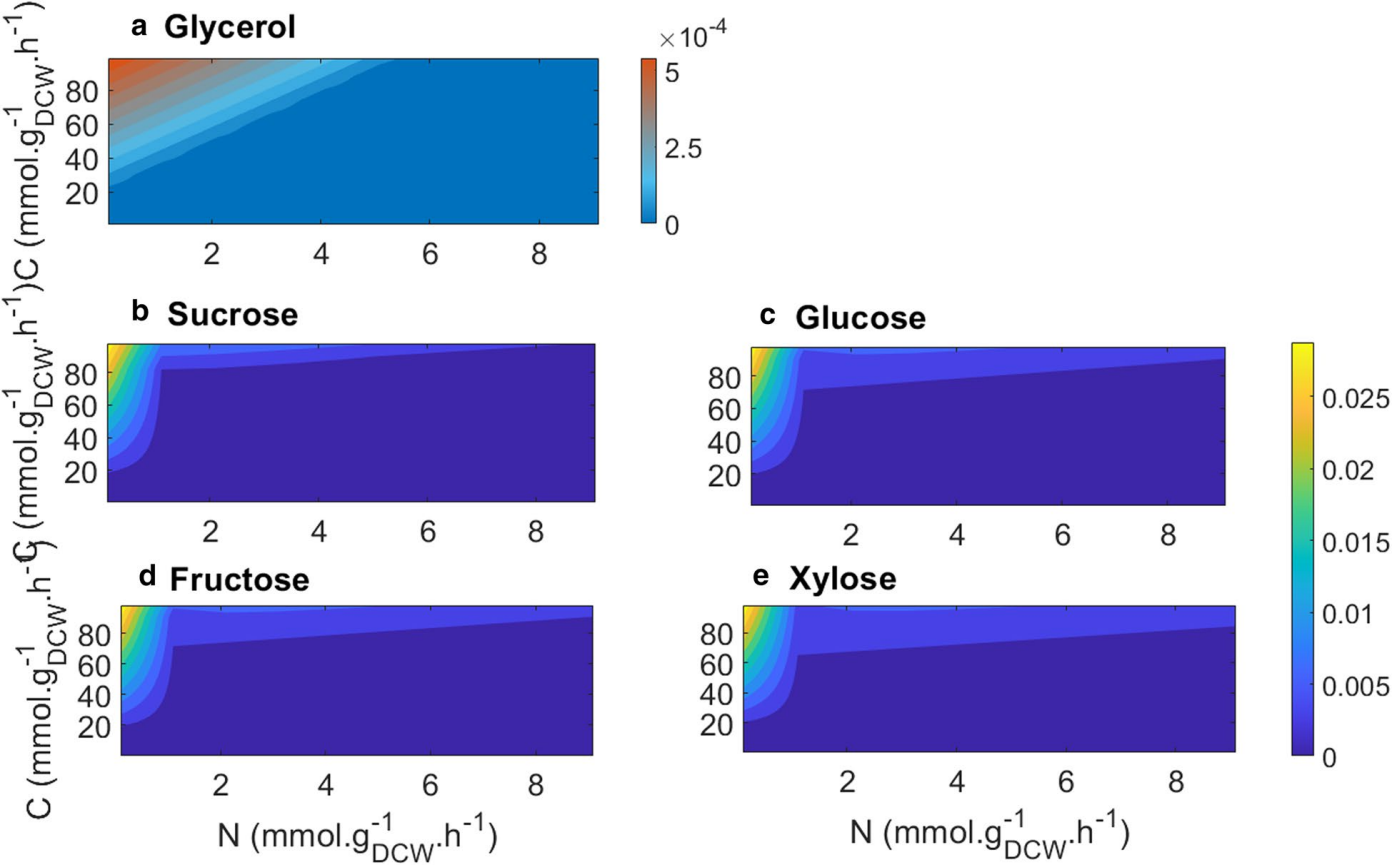
In silico lipid production rate when using different carbon sources. *X* and *Y* axis started from 0.1. The color bars indicate lipid production rate mmol. g_DCW_ ^−1^ h^−1^

As for growth, a similar trend was predicted for lipid production on different carbon sources (Fig. 6). For all tested carbon sources, the model predicted highest lipid production at high C/N ratios. Model prediction for lipid production in glycerol is noticeably different from that of other carbon sources. This is consistent with findings in [44] who reported a maximum growth rate and lipid production of *C. oleaginosus* ATCC 20509 on glycerol in a fed-batch fermentation mode at 16* g/l* glycerol and 0*.*27* g/l* NH_4_Cl, corresponding to a C/N ratio of 100 mol/mol.

The model predicted the highest lipid production rate in sucrose, glucose, fructose, and xylose. Glycerol yielded lower lipid production rate. Similar to growth, literature also captured contrasting findings on lipid production on different carbon sources. Across various carbon sources, xylose was found the most suitable sugar source for lipid yield in batch and chemostat cultures [22]. On the other hand, a lower lipid production on xylose compared to glucose were reported in other studies [25, 45]. This disagreement between studies can be due to influence of other factors such as temperature, oxygen and fermentation mode [11].

### Acetyl-CoA source for lipid production in C. oleaginosus ATCC 20509

Lipid synthesis requires a constant supplement of fatty acid and fatty acid synthesis in turn requires a continuous supplement of acetyl-CoA [32]. In non-oleaginous yeast such as *S. cerevisiae*, the main source of acetyl-CoA is the ligation of acetate and coenzyme A by acetyl-coA synthase [32]. Oleaginous yeast such as *Y. lipolytica*, do not have the gene encoding for acetyl-CoA synthase [32].

**Fig. 7:**
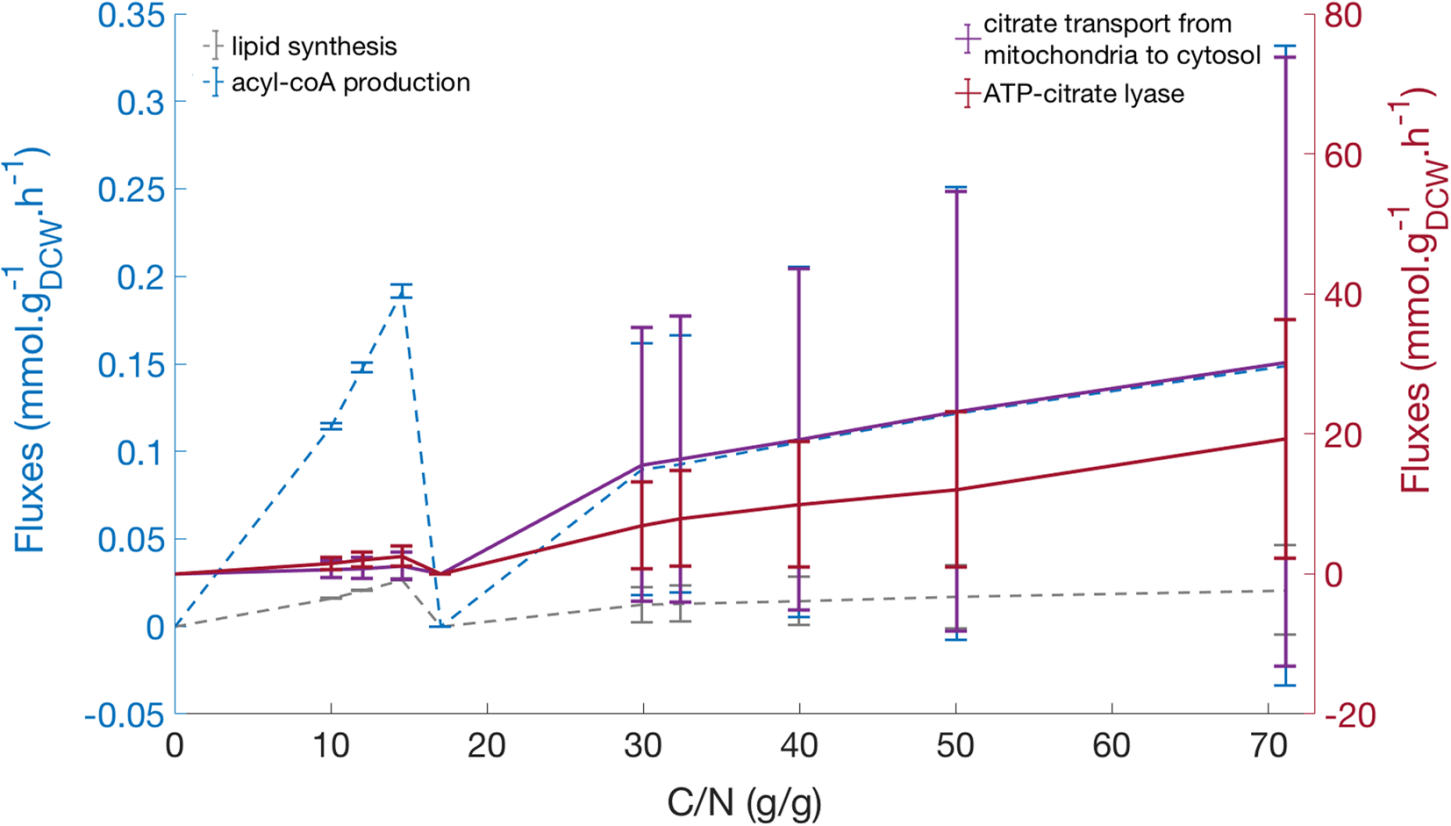
In silico flux analysis of acetyl-CoA source for lipid synthesis. The citrate transport from mitochondria to cytosol and ATP-citrate lyase which catalyzes the reaction ATP + citrate + Coenzyme-A acetyl-CoA + oxaloacetate + *P*_i_ + ADP in cytosol follow the red Y-scale; acyl-coA production (an artificial reaction represents acyl-coA Pool in the model); and lipid synthesis (an exchange reaction of lipid) follow the blue *Y*-scale. Bars indicate the standard errors of means of fluxes through each reaction. The C/N (g/g) refers to the ratio between the uptake rates of carbon and nitrogen sources

The main source for acetyl-CoA in oleaginous yeasts is believed to be the cleavage of citrate to release acetyl-CoA and oxaloacetate in the cytosol by ATP:citrate lyase [32]. This implies that there is a continuous export of citrate from the mitochondria to the cytosol. Our model, under assumed chemostat cultivation conditions, also predicts this. The flux of the citrate transport reaction increases positively with ATP:citrate lyase whose flux also increases sharply after passing C/N ratio of 10 g/g (Fig. 7). Fluxes through acetyl-coA pool and lipid synthesis reaction also surged after passing the same C/N ratio (Fig. 7). The large standard deviations in Fig. 7 represent alternative flux distributions that are compatible with the set constraints. This variability reflects both the metabolic flexibility of this organism and the lack of sufficient data to fully constrain the model, a common problem in GEM model analysis.

As reported in [46], after passing the critical C/N of 11 g/g, when the nitrogen concentration is limiting further growth, the yeast starts to accumulate more lipids. In order to sustain cellular functioning, the cell degrades AMP to inosine monophosphate and ammonium ions [3, 32]. A decreased AMP concentration in turn down-regulates the activity of isocitrate dehydrogenase [3, 32, 47, 48]. This enzyme converts citrate to isocitrate. Its down-regulation, therefore, leads to the accumulation of citrate in mitochondria. Accumulated citrate is then exported to cytosol where it is hydrolysed to acetyl-CoA and oxaloacetate by ATP:citrate lyase [3, 32]. This process provides more acetyl-CoA for fatty acid synthesis which further enhances lipid production in the cell [3]. Although FBA analysis does not account for regulation, the same trend was observed in our simulations, that clearly indicate the association between increased flux through ATP:citrate lyase reaction and lipid production (Fig. 7). Furthermore, model simulations show no alternative lipid production pathway as in silico growth is inhibited when simulating a knock out of this enzyme. Our model suggests the ATP-citrate lyase reaction as the main source for acetyl-CoA suggesting that overexpression of ATP-citrate lyase enzyme could help to further improve lipid production. This strategy has been successfully implemented in *Y. lipolytica* [49].

### Lipid metabolism regulation

**Fig. 8:**
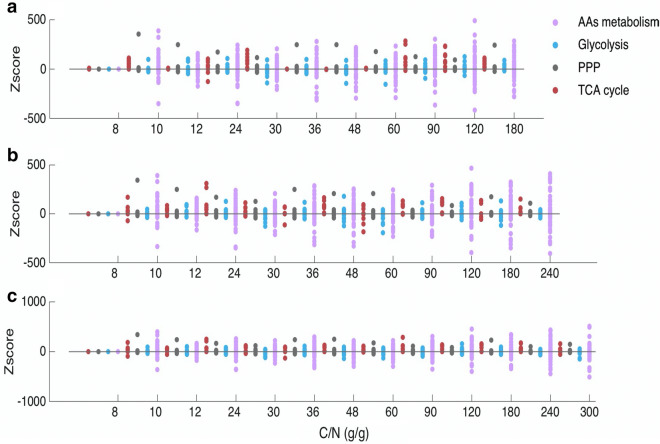
In silico flux changes in *C. oleaginosus* ATCC 20509 at different C/N ratio with **a** 16 g carbon; **b** 24 g carbon; **c** 32 g carbon using glycerol as a sole carbon source. A C/N ratio of 6 g/g was used as reference point to calculate *Z*-score for each C/N ratio. The C/N (g/g) refers to the ratio between the uptake rates of carbon and nitrogen source. *Z*-score > 0 indicates an increase in flux compare to that at reference point; *Z*-score < 0 indicates a decrease in flux compared to that at reference point. PPP–pentose phosphate pathway; AAs metabolism–amino acids metabolism. Each dot in the graph represents a reaction in the corresponding pathway

The effect of nitrogen limitation on lipid production was studied by analyzing the effect of the C/N ratio on (i) the in silico flux distribution and (ii) the transcriptional landscape of *C. oleaginosus* ATCC 20509 grown on glycerol.

i. *In silico flux distribution:* We tested lipid production at different C/N ratios while keeping the carbon concentration constant at either 16, 24 or 32 g/g DCW (Fig. 8) as for the same C/N ratio the absolute amount of carbon supplied has been shown to greatly affect lipid production [8]. The model predicted that for the higher C/N ratios, more carbon is required to sustain lipid production. With 16 g/g DCW carbon no lipid could be produced at a C/N ratio of 240 (g/g). Likewise, with 24 g carbon, no lipid formation was predicted at a C/N ratio of 300 (g/g). Only with 32 g/g DCW carbon, lipid accumulated at the complete range of C/N ratio’s tested.

For the three tested carbon concentrations, the same trends in flux distribution were obtained (Fig. 8). When increasing the C/N ratio, a majority of reactions in TCA and PPP have their fluxes increased. This could be due to the high demand of reducing power, i.e. NADPH, of lipid production. Fluxes through glycolysis are greatly diverse when changing the glycerol concentration. Reactions related to glucose catabolism such as hexokinase (d-glucose:ATP) and glucose-6-phosphate isomerase (PGI) have their fluxes reduced. Down regulation of PGI was reported to lead to the accumulation of intracellular sugar which is later converted to lipid in the nitrogen-depletion stage [41]. Downstream reactions in glycolysis such as 6-phosphofructo2-kinase, pyruvate kinase and acetyl-CoA synthetase have their fluxes increased. Upregulation of these enzymes can be a result from a high demand of precursors for lipid accumulation.

As already mentioned in [50] we also observed flux fluctuations in amino acid metabolism (Fig. 8). Fluxes through enzymes in amino acid degradation pathways, i.e. argininosuccinate lyase, L-hydroxyproline dehydrogenase (NAD), and L-glutamate 5-semialdehyde dehydratase increase at a high C/N ratio. This is expected since upon nitrogen limitation, amino acid degradation provides an alternative nitrogen source. Reactions in amino acids synthesis such as glutamine synthetase and ornithine decarboxylase, on the other hand, had their fluxes reduced.

ii. *Nitrogen limitation induced transcriptional changes:* RNAseq data were obtained from *C. oleaginosus* ATCC 20509 when growing in a glycerol medium with an initial C/N (g/g) ratio of 28 and 2.8, respectively. At the time of sampling, nitrogen was significantly depleted when the high C/N ratio was used (Table 5). There were 7272 genes expressed in high C/N ratio medium and 7246 genes expressed in low C/N ratio medium (> 50 Counts Per Million). When comparing low C/N ratio to high C/N ratio medium, 75 genes were found to be up-regulated and 26 were down-regulated (see Additional file 5). Interestingly, the majority of these genes code for unknown protein functions. No genes involved in primary metabolism were found to have significant different expression level in either low or high C/N ratio.

**Table 5:** Glycerol and NH4Cl levels obtained from HPLC analysis and NH4 chemical analysis

	OD 650		glycerol (g/L)		NH4Cl (g/L)		C/N (mol/mol)	CDW^a^ (mg/ 50 ml)
	*T* = 0 h	*T* = 18 h	*T* = 0 h	*T* = 18 h	*T* = 0 h	*T* = 18 h	*T* = 18 h	
A1	2.75	12.2	20.5	11.3	1.1	0.022	896	404
A2	2.9	11.8	20.5	11.4	1.1	0.015	1325	365
B1	2.8	10.4	10.2	1.6	4.7	2.9	0.96	445
B2	2.85	10.4	10.2	2.5	4.7	3.6	1.2	445

^a^Cell dry weight

In response to nitrogen starvation the gene expression levels of many genes in the lipid synthesis pathway were reported to fluctuate in *Y. lipolytica* [51], in contrast Kerhoven et al. [50] reported no significant change in transcription level of these genes under nitrogen limitation. Using xylose as carbon source the Acetyl-CoA carboxylase (ACC) gene was found to be upregulated in *Trichosporon oleaginosus* strain IBC0246 under nitrogen limitation [52] and the same authors also reported significant upregulation of fatty acid synthetase (FAS1 and FAS2), malic enzyme and ATP-citrate lyase (ACL) under these conditions.

In our case, upon growth in glycerol, RNAseq analysis showed no difference in transcription level of genes involved in lipid synthesis pathway in *C. oleaginosus* ATCC 20509. The model however, was able to predict lipid production at different C/N ratio qualitatively consistent with experimental data. This suggests that in glycerol *C. oleaginosus* ATCC 20509 lipid metabolism is not regulated at the transcriptional level. Pathway flux is controlled by simultaneous multisite modulation through action on a number of enzymes [53]. This suggests that other regulatory effects, such as regulation of translation or allosteric effects may dominate in *C. oleaginosus*. In *Lipomyees starkeyi*, an oleaginous yeast, and *Aspergillus niger*, a citric producing yeast, ATP:citrate lyase, the key enzyme in lipid synthesis is controlled by the energy charge and fatty acid acyl CoA esters [54]. While human ATP:citrate lyase activity has been reported to be regulated by in vitro allosteric effects via phosphorylation [55]. Little is know on the regulation of this enzyme in *C. oleaginosus*.

## Conclusions

In this study, we introduced the first GEM for *C. oleaginosus* ATCC 20509 and as such *i*NP636_*Coleaginosus*_ATCC20509 represents a valuable platform to integrate, interpret and combine many decades of experimental efforts since its first isolation from a dairy farm in 1978 [13, 56]. The model gave qualitative predictions at different C sources consistent with experimental data, highlighted the lipid production lifestyle of *C. oleaginosus* ATCC 20509 and pinpointed ATP-citrate lyase as a target to further improve lipid production. Analysis of RNAseq revealed that lipid production in *C. oleaginosus* ATCC 20509 in glycerol does not appear to be regulated at the transcriptional level.

*C. oleaginosus* is known to have a great potential for lipid production due to its efficient growth on inexpensive carbon sources such as glycerol. Our simulations show that its potential has not yet been fully explored and can be optimized further. The predictive accuracy of *i*NP636_*Coleaginosus*_ATCC20509 renders its great potential for future studies to guide metabolic engineering for the production of high-value industrial compounds such as polyunsaturated plant-like fatty acids.

## Materials and methods

### *C. oleaginosus ATCC* 20509 experimental data collection

The strain was cultivated in the same basal medium as described in [8] except for the glycerol and NH_4_Cl concentration which was adapted in order to achieve the chosen C/N ratio. A C/N ratio of 28 was obtained by adding 16 g/l glycerol and 1 g/l NH_4_Cl (medium A), while in other sample, 8 g/l glycerol and 5 g/l NH_4_Cl was added to make a C/N ratio of 2.8 (medium B). The C/N ratios were taken from [8], which shows *C. oleaginosus* grows at a C/N ratio of less than 5, and lipid production for a ratio between 20 and 40 carbon / nitrogen.

Two biological replicates for each condition were inoculated from a freshly prepared YPD-agar plate in 50 ml of YPD medium and grown O/N in a 100-ml Erlemeyer flask at 30 ^0^ C and 225 rpm. The culture was divided in two 25 ml portions and centrifuged (10 min. 300 rpm) to collect the cells. The cell pellets were resuspended in 30 ml medium A or medium B. 4 ml of the resuspended cells was used to start duplicate cultures in medium A and B which were incubated for 18 h at 30 °C and 225 rpm. Each culture was divided in two equal portions and the cells were harvested by centrifugation and the wet pellet frozen in liquid and used for RNA extraction.

We measured the concentration of glycerol and NH_4_Cl in the medium at the initial condition and at the sampling point (Table 5). Glycerol and NH_4_Cl were measured with HPLC analysis and NH4 chemical analysis, respectively.

### RNA extraction procedure

RNA was extracted using an acidic hot phenol extraction procedure. Briefly, the cell pellet was ground in liquid nitrogen and mixed with 4 volumes of pre-warmed (60 °C) phenol + extraction buffer (1% SDS, 10 mM EDTA, 0,2 M NaAc (pH 5)) after this 2 volumes of chloroform were added and mixed thoroughly. After centrifugation the buffer layer was washed once with chloroform. RNA was precipitated from the buffer layer by adding 8 M LiCl to and end concentration of 2 M. After centrifugation the pellet was washed once with 2 M LiCl and twice with 70% ethanol. The remaining pellet was resuspended in RNase-free water. Total RNA extract, RNA sequencing, and RNAseq data processing were performed as described in [8]. Samples were sequenced by NovoGene using Total RNA.

### RNAseq analysis

Raw read counts in two C/N ratios, 2.8 and 28 (mol C/mol N), were obtained with the RNA-seq aligner STAR (v2.6.0b) [57] using the parameter “–quantMode GeneCounts”, the public genome sequence MATS00000000.1 of *C. oleaginosus* ATCC 20509 and the GTF file obtained from BRAKER1. Read count data were then analyzed using DESeq2 [58] to identify genes that have different expression when changing the C/N ratio. Two biological replicates for each condition were provided. The statistical significance of gene expression differences was evaluated using a false discovery rate (FDR) < 0.05 and |log_2_ (fold change)|≥ log_2_ 1*.*5 as a threshold.

### Genome sequence

The genome sequence MATS00000000.1 from *Cutaneotrichosporon oleaginosus* ATCC 20509 reported by [28] was annotated and used to build the model. The genome sequence has 19.86 Mbp and a GC content of 60.7%.

### Genome annotation

Unsupervised RNA-Seq-based gene prediction of *C. oleaginosus* ATCC 20509 was performed with BRAKER1 v1.10 [29] in combination with HISAT2 (v2.1.0) [59] using all the RNAseq datasets combined.

The genome, predicted gene structures and their proteins sequences were directly stored in the SAPP semantic (RDF) database [60] using the GBOL ontology [61]. Protein signature prediction was done with a standalone version of InterProScan v5.24.64.0 [62] using the default databases. EnzDP [63] was used to assign EC numbers to Proteins. This is with a confidence score cut-off of 0.2. Both tools were used in direct interaction with the previously mentioned SAPP database. Construction of *i*NP636_*Coleaginosus*_ATCC20509 model.

### Software environment

The model was read, modified and analyzed in MATLAB (version R2015b) [64], using COBRA toolbox 3.0 [65] and GLPK [66] as a linear solver.

### Construction of the draft model

A draft model was constructed using the scaffold-based method described in [37]. A GEM of *Y. lipolytica*, considered as a model for oleaginous organism [2, 32] was chosen as a reference scaffold. There are 5 published models for *Y. lipolytica* iNL895 [37], iYL619 [67], iMK735 [68], iYALI4 [50], iYLI647 [69]. *Y. lipolytica* iNL895 model [37] was used as a scaffold because it contains the most reactions and genes and was also constructed based on the *S. cerevisiae* model, iIN800 [70] which was specialized for lipid synthesis.

To find ortholog proteins from *Y. lipolytica* to *C. oleaginosus* ATCC 20509, the enzyme-coding-genes obtained from *Y. lipolytica* iNL895 model were functionally annotated in the same manner as *C. oleaginosus* ATCC 20509 and stored in the SAPP database. A combination of the protein signatures, EC prediction, BLAST and manual curation was used to find the orthologues.

If an ortholog gene was found in *C. oleaginosus* ATCC20509, the associate reaction in the scaffold iNL895 was kept. In addition, exchange and non-enzymatic transport reactions for the medium were kept. Spontaneous and growth essential orphan reactions from the scaffold were also preserved. This step resulted in a draft model for further curation.

### Curation of the draft model

In order to build a working GEM the draft model expanded and refined in the following manner:

i. The lipid synthesis pathway was curated based on KEGG [71], literature [3–5, 32] as well as experimental data in [8, 44].
ii. The central metabolic network, including glycolysis, pentose phosphate pathway and TCA cycle were manually curated based on literature [72, 73].
iii. Growth-associated maintenance energy (GAM) was adopted from *Y. lipolytica* model, iNL895. Non-growth associated maintenance energy in *C. oleaginosus* ATCC 20509 is known to be relatively low in comparison with other yeasts [8], in the model this value was set as 1 mmol · *gDCW* − 1 · *h* − 1.
iv. The draft model was further curated by removing gaps, irrelevant reactions and infeasible energy production cycles. Method described in [74] was employed to identify infeasible energy production cycles in our model. In short, we added energy dissipation reactions for ATP, CTP, GTP, UTP, NADH, NADPH, FADH2 and proton with unconstrained bounds. Fluxes through all network reactions, except the added energy dissipation reactions were constrained to range [− 1,1] for reversible and [0,1] for irreversible reactions. No uptake nutrients were allowed. Each energy dissipation reaction was maximized to identify the presence of infeasible loops.

### Development of a condition-specific biomass function

The four major macro-molecules of living cells are proteins, carbohydrates, nucleic acids and lipids [75]. The ratio between them are assumed to be different in different conditions. We assumed lipid, protein and carbohydrate makeup 95% of the cell dry weight. Depending on the C/N ratio in the medium, the ratio between them will vary. Nucleic acids and other cofactors and mineral only make up a small fraction of the biomass, and kept constant. Using data from literature, we parametrized the relationship between the biomass and carbohydrates, proteins and lipids under nitrogen starvation using:

$$ 0.{\text{11biomassCarbohydrate}}\, + \,{\text{biomassProtein}}\, + \,{\text{biomasstotalLipid}}\, + \,0.0{\text{5biomassother}}\, = \,{\text{biomass}}. $$

This assumes that under nitrogen starvation, 11% of the cellular biomass corresponds to carbohydrates, 5% to nucleic acids and other components and the remaining fraction correspond to proteins and total lipids. We used the experimental data in [25] to model the C/N ratio in the media and lipid accumulation using a quadratic regression (Fig. 3) with a correlation coefficient of 0.98. This enables the estimation of the contribution of lipids *w*_*TL*_ to the biomass. For this, C and N uptake rates were used to compute the ratio between both components as we assumed a simulation scenario (chemostat) where no net accumulation of either one happens. Using this approach, we can generate specific biomass reaction at any C/N ratio using carbon source and nitrogen source uptake rates as the sole inputs. Details regarding components and their coefficients in the biomass reaction at normal condition, i.e. when there is no nitrogen depletion, can be found in Additional file 6. Finally, the biomass equation was standardized to have a molecular weight of 1 · *g/mmol*.

The main lipid building-blocks are fatty acid residues. The majority of fatty acid in *C. oleaginosus* ATCC 20509 is oleic acid (C18:1) [4, 5]. When growing on glucose, the composition of main fatty acids in *C. oleaginosus* ATCC 20509 are 25% hexadecanoic (C16:0), 10% octadecanoic acid (C18:0), 57% oleic acid (C18:1), and 7% linoleic acid (C18:2) [4, 5]. As specific information about each fatty acid in lipid molecules is not available for *C. oleaginosus* ATCC 20509, in the model, an artificial acyl-CoA pool for lipid synthesis was formulated. A reaction representing the acyl-CoA pool was introduced:

$$ 0.{24952}C_{{{16}:0}} \, + \,0.0{96712}C_{{{18}:0}} \, + \,0.{55233}C_{{{18}:{1}}} \, + \,0.0{67963}C_{{{18}:{2}}} \, \to \,Acyl\, - \,CoA_{pool} . $$

Coefficients of fatty acids in the acyl-CoA pool reaction represent their weight percentages in the lipid of *C. oleaginosus* ATCC 20509 according to data in [4, 5].

### Growth simulation

Model accuracy was validated using flux balance analysis (FBA) implemented in COBRA Toolbox 2.0.6 [76] in MATLAB environment. Minimum defined medium was used. Unlimited uptake rates of CO_2_, H_2_O, H^+^, O_2_, Iron^2+^, phosphate, potassium, sodium, sulphate, and NH_4_ were allowed. This entailed setting the lower bounds of the corresponding exchange reactions to − 1000, as we used the usual convention of writing the exchange reactions in such way that production corresponds to positive fluxes and consumption to negative ones. These constraints were kept for all simulations.

The gold standard validation technique in GEMs is to compare model prediction to experimental data. In silico growth simulation in the presence of different carbon sources was carried out, for this uptake rate of the corresponding carbon source was constrained to − 10 mmol · *g*_*DCW*_^−1^ · *h*^−1^. Biomass reaction with experimentally determined content at nitrogen abundant condition was used.

Investigation of lipid synthesis in *C. oleaginosus* ATCC 20509.

### Simulations of growth and lipid production

We conducted in silico experiments to assess the effect of C/N ratio on lipid production in *C. oleaginosus* ATCC 20509. To compare our prediction with simulation from the response surface method [25], we mimic the experimental setup in [25].

To generate different C/N ratios, C mmol and N mmol were calculated from the data in [25] where nitrogen was set up in the range of [0.1:0.01:0.8] g, carbon was in [1.5: 0.05: 8.5] g with urea and glucose as nitrogen and carbon source, respectively. We assumed a constant uptake of carbon and nitrogen. As reported in [46] after passing a critical C/N of 12.83 (mol/mol) or 11 (g/g) the biomass reaches the maximum value of 0.20 h^−1^. Thus, to simulate lipid production we fixed the growth rate for subsequent optimizations. If the in silico growth rate at the tested C/N ratio was higher than 0.2 h^−1^, we fixed the biomass lower bound and upper bounds to [0*.*2·0*.*9*,*0*.*2]. For growth rates smaller than 0.2 h^−1^, we fixed the biomass to the maximal predicted values at the corresponding C/N ratio. A specific biomass reaction for each C/N ratio was used. Lipid formation happens when the cell was subjected to sudden depletion of other nutrients such as nitrogen after growing maximally [5]. To mimic this process, in our simulation, biomass function was constrained to the set values and exchange reaction of lipid body was maximized. We did not constrain biomass when simulating for growth.

To study effects of different carbon sources on growth and lipid production, lower bound and upper bound of each exchange reaction for glucose, fructose, xylose, sucrose, ethanol and glycerol was constrained in each study. To generate different C/N ratio the uptake rate was increased gradually in the range of − [0.1: 5: 100] *mmol* · *g*_*DCW*_^−1^ · *h*^−1^ for carbon source and of − [0.1: 1: 10] *mmol* · *g*_*DCW*_^−1^ · *h*^−1^ for nitrogen.

### Sampling the solution space when shifting C/N ratio

To study how flux distribution change when changing C/N ratio, we sampled the solution space at steady state for each C/N ratio. Based on [25, 46] we selected the C/N ratio as [6,8,10,12,24,30,36,48,60,90,120,180,240]. To study the effect of carbon concentration on lipid synthesis we simulated lipid production at 3 different C (g) as [16, 24, 32] for the same C/N range. Minimal medium was used. The solution space at steady state for each C/N ratio when optimizing for lipid production with constrained biomass (see Section “Simulations of growth and lipid production”) was sampled using gpSampler [77] implemented in COBRA toolbox 2.0.6 [76]. The sample was taken for 5000 sample points with no bias, “maxtime” was 10 min, “maxsteps” was set to 10^10^ and 1 thread was used. Sampling results were analyzed as described in [78]. In short, means and standard deviations were calculated from the sampling results to obtain *Z* scores for each reaction in the central metabolic network. A C/N ratio of 6 (g/g) was used as the reference point to calculate *Z* scores for fluxes in other C/N ratio.

To study the main source of acetyl-coA for lipid synthesis in *C. oleaginosus* ATCC 20509, we sampled the solution space at steady state for each C/N ratio. The C/N ratio data from experiment in [46] were used for the simulation. To mimic their experimental setup, uptake rate of nitrogen, in form of urea, was fixed at − 25 mmol · *g*_*DCW*_^−1^ · *h*^−1^. Carbon was gradually increased to generate the desired C/N ratio.

## Supplementary Information

**Additional file 1.*** C. curvartum* ATCC 20509 genome annotation.

**Additional file 2.** Details on lipid synthesis pathways.

**Additional file 3.** *C. oleaginosus* and* Y. lipolytica *orthologs

**Additional file 4.** Condition-specific biomass generation.

**Additional file 5.** List of up and down-regulated genes under high and low C/N condition.

**Additional file 6.** Biomass component at glucose and unlimited nitrogen condition.

**Additional file 7.** *i*NP636_Coleaginosus_ATCC20509 model.

## Data Availability

RNAseq data generated and analyzed during this study were deposited in the European Nucleotide Archive (ENA) repository under accession number PRJEB34237 (url). The model *i*NP636_*Coleaginosus*_ATCC 20509 was deposited in BioModels [79] and assigned the identifier MODEL2004170002 and is also provided in this paper as Additional file 7. All other data that support the findings of this study can be found in Additional files 1–7.

## Abbreviations

GEM: Genome-scale metabolic model
TAG: Triacylglycerol
ACL: ATP-citrate lyase
ACC: Acetyl-CoA carboxylase
FAS: Fatty acid synthetase
