# Supplementary material for: Genome-scale metabolic modeling underscores the potential of Cutaneotrichosporon oleaginosus ATCC 20509 as a cell factory for biofuel production
Source: Biotechnol Biofuels. 2021 Jan 6;14:2. doi: 10.1186/s13068-020-01838-1 (PMC7788717; doi:10.1186/s13068-020-01838-1)
Supplement: Supplementary file 2 — Additional file 2. Details on lipid synthesis pathways. [file 13068_2020_1838_MOESM2_ESM.pdf]

Lipid synthesis pathways

C. oleaginosus ATCC 20509 metabolizes sugars by using standard central metabolic pathways including glycolysis, pentose phosphate pathway and the citric acid (TCA) cycle (Figure 1). These pathways provide the precursors and energy required for for lipid biosynthesis. Lipid biosynthesis can be divided into three steps: formation of fatty acids, synthesis of triacylglyceride (TAG), and synthesis of phospholipids.

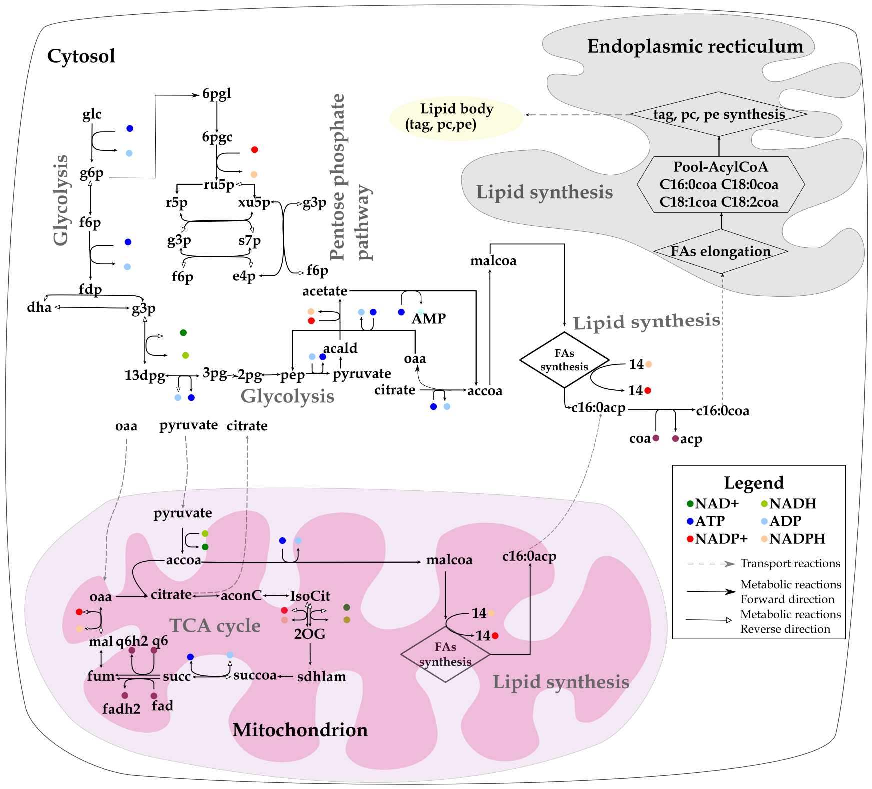

Figure 1 Central carbon metabolism and lipid synthesis pathway in *C. oleaginosus* ATCC 20509. 13dpg- 3-Phospho-D-glyceroyl phosphate; **2OG** - 2-Oxoglutarate; **2pg** - D-Glycerate 2-phosphate; **3pg** - 3-Phospho-D-glycerate; **acald** - acetaldehyde; **accoa** - acetyl-CoA; **aconC** - cis-Aconitate; **acp** - acyl carrier protein; **c16:0acp** - Hexadecanoyl acyl carrier protein; **c16:0coa** - Hexadecanoyl-coa; **c18:0coa** - Octadecanoyl-coa; **c18:1coa** - oleoyl-CoA ; **c18:2coa** - linoleoyl coA; **coa** - coenzyme A; **dha** - dihydroxyacetone ; **f6p** - fructose-6-phosphate; **fdp** - D-Fructose 1,6-bisphosphate; **fum** - fumarate; **g3p** - glyceraldehyde 3-phosphate; **g6p** - glucose-6-phospahte; **glc** - glucose; **IsoCit** - Isocitrate; **mal** - malate; **malcoa**- malonyl-CoA; **oaa** - oxaloacetate; **pc** - phosphatidylcholine; **pe** - phosphatidylethanolamine; **pep** - phosphoenolpyruvate; **q6** - Ubiquinone 6; **q6h2**- Ubiquinol 6; **sdhlam** - S Succinyldihydrolipoamide ; **succ** - succinate; **succoa** - succinyl-CoA ; **tag** - triacylglycerol.

Formation of fatty acids

In yeasts, fatty acids can derive from either a *de novo* synthesis pathway, from hydrolysis of complex lipids and delipidation of proteins or from hydrolysis of external fatty acids sources [1]. *De novo* fatty acid synthesis mainly occurs in the cytosol [2], and in some cases, in the mitochondrion [3]. The process is catalyzed by the multi-enzyme fatty acid synthetase complex (FAS) [2]. We found multiple genes, g2870.t1, g5734.t1, g570.t1 and g5733.t1, that together encode this enzyme com-

plex in *C. oleaginosus* ATCC 20509. The overall process of lipid synthesis can be simplified as follow:

- **ATP- citrate lyase (ACL)**. Citrate + ATP → oxaloacetate + acetyl-CoA + ADP + P_i
- **Acetyl-CoA Carboxylase (ACC)**. acetyl-coA + CO_2 + ATP → malonyl-CoA + ADP + P_i
- **Fatty acid synthetase (FAS)**. acetyl-CoA + 7 malonyl-CoA + 14 NADPH + 14 H^+ → palmityl-CoA + 14 $NADP^+$ + 7 CoA + 7 CO_2

To sustain lipid synthesis, a constant supply of fatty acids is required [4]. This process in turn requires a continuous supply of acetyl-CoA [4]. For this oleaginous yeast species [5] typically have a cytosolic ATP :Citrate Lyase [6], in *C. oleaginosus* ATCC 20509, encoded by g3238.t1, that catalyzes the cleavage of citrate to yield acetyl CoA and oxaloacetate.

The next step in *de novo* fatty acid synthesis is the condensation of an acetyl-CoA and a bicarbonate anion to generate malonyl-CoA [2] and gene g570.t1 was predicted to encode the required acetyl-CoA carboxylase.

Malonyl-CoA is then converted to malonyl-ACP by malonyl-CoA ACP transacylase (g5734.t1 and g5733.t1) [7], followed by a series of elongation reactions. For each step of the carbon chain elongation reaction, two molecules of NADPH are required for FAS. NADPH can come from the malic enzyme, which is found in the mitochondrion of most of oleaginous microorganism in the malate dehydrogenase carboxylase reaction [4]: S-Malate + $NADP^+$ → pyruvate + NADPH + CO_2 . In *C. oleaginosus* ATCC 20509 two genes encoding this enzyme g1447.t1 or g6387.t1 are present.

Synthesis of triacylglyceride

The process of triacylglyceride (TAG) synthesis can be split into 3 steps: starting with the formation of phosphatidic acid (PtdOH) from glycerol-3-phosphate either through the glycerol-3-phosphate or the dihydroxyacetone phosphate pathway [3, 8, 9] .

- Formation of PtdOH catalyzed by two different acyltransferase encoded by g1820.t1 and g6923.t1 respectively.
  acyl-CoA + glycerol 3-phosphate ⇔ 1-acyl-sn-glycerol 3-phosphate + coenzyme A + 2 H^+
  1-acyl-sn-glycerol 3-phosphate + acyl-CoA ⇔ coenzyme A + phosphatidate
- Formation of diacylglyceride (DAG) reaction catalyzed by phosphohydrolase enzyme (g920.t1)
  Phosphatidate + H2O ⇔ diglyceride + phosphate + 2 H^+
- Formation of TAG catalyzed by acyltransferase enzyme (g561.t1)
  Acyl-CoA + diglyceride ⇔ coenzyme A + triglyceride

Phospholipid synthesis

CDP-DAG pathway starts with the synthesis of liponucleotide CDP-DAG from phosphatidic acid and cytidine triphosphate (CTP) in the Endoplasmic recticulum (ER) [4, 10]. This reaction is catalyzed by CDP-diacylglycerol synthase enzyme (g6796.t1). CDP-DAG is then converted to PS by CHO1-encoded phosphatidylserine synthase (g3238.t1). PS is then transported to the mitochondrion, where it is

further decarboxylated to produce PE [10, 11, 12] by the enzyme encoded from g6020.t1. Transport of lipids from the ER and mitochondria is often considered via contact sites without long-distance through the cytosol [13, 10, 14]. However, a recent study showed that PS is transported to the mitochondrion via a cytosolic factor [11]. PE is transported to other cellular locations including ER to continue the synthesis of phospholipid [4, 10]. PC, the final product of the pathway is synthesized from PE through a series of methylation reactions. PC and PE can also form in the Kennedy pathway via two branches CDP-choline and CDP-ethanolamine, respectively [10]. The two branches start with external Ethanolamine or Choline, which need to be supplied in the culture medium [10]. Ethanolamine and Choline are phosphorylated in the cytosol with ATP to produce ethanolamine-P and choline-P, respectively. They are then further phosphorylated with CTP and incorporate DAG to produce PE or PC, respectively [15]. The later two steps are associated with enzymes in the nuclear/ER membrane [16]. In the abscence of ethanolamine and/or choline in the growth medium, CDP-DAG is the main route to synthesize PE and/or PC [16].

Author details

References

1. Tehlivets, O., Scheuringer, K., Kohlwein, S.D.: Fatty acid synthesis and elongation in yeast. Biochimica et Biophysica Acta (BBA)-Molecular and Cell Biology of Lipids **1771**(3), 255–270 (2007)
2. Beopoulos, A., Nicaud, J.-M., Gaillardin, C.: An overview of lipid metabolism in yeasts and its impact on biotechnological processes. Applied microbiology and biotechnology **90**(4), 1193–1206 (2011)
3. Klug, L., Daum, G.: Yeast lipid metabolism at a glance. FEMS yeast research **14**(3), 369–388 (2014)
4. Fakas, S.: Lipid biosynthesis in yeasts: A comparison of the lipid biosynthetic pathway between the model nonoleaginous yeast saccharomyces cerevisiae and the model oleaginous yeast yarrowia lipolytica. Engineering in Life Sciences **17**(3), 292–302 (2017)
5. Rodriguez, S., Denby, C.M., Vu, T.v., Baidoo, E.E., Wang, G., Keasling, J.D.: Atp citrate lyase mediated cytosolic acetyl-coa biosynthesis increases mevalonate production in saccharomyces cerevisiae. Microbial cell factories **15**(1), 48 (2016)
6. Ratledge, C., Wynn, J.P.: The biochemistry and molecular biology of lipid accumulation in oleaginous microorganisms. Advances in applied microbiology **51**, 1–52 (2002)
7. Beld, J., Lee, D.J., Burkart, M.D.: Fatty acid biosynthesis revisited: structure elucidation and metabolic engineering. Molecular BioSystems **11**(1), 38–59 (2015)
8. Rismani-Yazdi, H., Haznedaroglu, B.Z., Bibby, K., Peccia, J.: Transcriptome sequencing and annotation of the microalgae dunaliella tertiolecta: pathway description and gene discovery for production of next-generation biofuels. BMC genomics **12**(1), 148 (2011)
9. Athenstaedt, K.: Neutral lipids in yeast: synthesis, storage and degradation. In: Handbook of Hydrocarbon and Lipid Microbiology, pp. 471–480. Springer, ??? (2010)
10. Flis, V.V., Daum, G.: Lipid transport between the endoplasmic reticulum and mitochondria. Cold Spring Harbor perspectives in biology **5**(6), 013235 (2013)
11. Junker, M., Rapoport, T.A.: Involvement of vat-1 in phosphatidylserine transfer from the endoplasmic reticulum to mitochondria. Traffic **16**(12), 1306–1317 (2015)
12. Kannan, M., Sivaprakasam, C., Prinz, W.A., Nachiappan, V.: Endoplasmic reticulum stress affects the transport of phosphatidylethanolamine from mitochondria to the endoplasmic reticulum in s. cerevisiae. Biochimica et Biophysica Acta (BBA)-Molecular and Cell Biology of Lipids **1861**(12), 1959–1967 (2016)
13. Toulmay, A., Prinz, W.A.: Lipid transfer and signaling at organelle contact sites: the tip of the iceberg. Current opinion in cell biology **23**(4), 458–463 (2011)
14. Scharwey, M., Tatsuta, T., Langer, T.: Mitochondrial lipid transport at a glance. The Company of Biologists Ltd (2013)
15. Gibellini, F., Smith, T.K.: The kennedy pathway—de novo synthesis of phosphatidylethanolamine and phosphatidylcholine. IUBMB life **62**(6), 414–428 (2010)
16. Henry, S.A., Kohlwein, S.D., Carman, G.M.: Metabolism and regulation of glycerolipids in the yeast saccharomyces cerevisiae. Genetics **190**(2), 317–349 (2012)
